# When Type 2 Diabetes Isn’t Type 2: Latent Autoimmune Diabetes in a Lean, Highly Physically Active Adult

**DOI:** 10.7759/cureus.102964

**Published:** 2026-02-04

**Authors:** Saraswathi Saiprasad, Narayana Swamy

**Affiliations:** 1 Endocrinology, Diabetes, and Metabolism, Baylor Scott & White Health, Fort Worth, USA; 2 Rheumatology, Baylor Scott & White Health, Fort Worth, USA

**Keywords:** autoimmune diabetes, automated insulin delivery, glycemic variability, hypoglycemia, lada, latent autoimmune diabetes in adults (lada)

## Abstract

Latent autoimmune diabetes in adults (LADA) is frequently misclassified as type 2 diabetes mellitus (T2DM), leading to delayed diagnosis and suboptimal management. We report a lean, highly physically active adult engaged in sustained, high-intensity physical activity as part of his occupation, in his early 70s, with a 30-year history of presumed T2DM who was referred for endocrine evaluation because of persistent glycemic instability despite insulin therapy. He reported frequent symptomatic episodes of both hyperglycemia and hypoglycemia, resulting in significant occupational stress, as maintenance of physical fitness and body weight was essential for continued employment. Hemoglobin A1c (HbA1c) values remained persistently elevated, ranging from 7.8% to 9.6% (reference range: 3.8%-5.6%), with marked glycemic variability and failure to achieve stable control. Given his low body mass index (BMI) of 20 kg/m² (reference range: 18.5-24.9 kg/m²) and pronounced glucose fluctuations, evaluation for autoimmune diabetes was pursued. Laboratory testing demonstrated markedly elevated glutamic acid decarboxylase (GAD65) antibodies (204 IU/mL; reference range <5 IU/mL) and a low C-peptide level (0.54 ng/mL; reference range 1.10-5.50 ng/mL), confirming LADA with advanced beta-cell failure. Management was transitioned from empiric multiple daily insulin injections to a tubeless automated insulin delivery (AID) system integrated with continuous glucose monitoring (CGM). Over longitudinal follow-up, glycemic control improved substantially, with reduced variability and minimal hypoglycemia. At the most recent follow-up, HbA1c was 6.8% (reference range: 3.8%-5.6%). CGM demonstrated a mean glucose of 153 mg/dL, consistent with CGM-derived targets corresponding to an estimated HbA1c <7% (goal <154 mg/dL), with 74% time-in-range (70-180 mg/dL) and less than 2% time below range (<70 mg/dL). This case highlights key clinical clues for recognizing LADA in adults initially labeled as having T2DM and underscores the importance of timely diagnosis and technology-enabled therapy, particularly in individuals with occupationally demanding physical activity.

## Introduction

Latent autoimmune diabetes in adults (LADA) is an autoimmune form of diabetes characterized by adult onset, the presence of islet autoantibodies (ICAs), and a gradual decline in pancreatic beta-cell function. Although immunologically related to type 1 diabetes mellitus (T1DM), LADA often presents clinically like type 2 diabetes mellitus (T2DM), with preserved insulin secretion at diagnosis and a period of partial insulin independence. Because of this overlap, LADA is frequently misclassified as T2DM, leading to delayed recognition of insulin deficiency and suboptimal treatment strategies.

Epidemiologic studies suggest that LADA accounts for approximately 2% to 12% of adult-onset diabetes, with prevalence varying by population and testing practices [1]. Importantly, an estimated 5% to 10% of adults initially diagnosed with T2DM may have underlying autoimmune diabetes that remains unrecognized when ICA testing is not routinely performed [2-4]. Such misclassification has significant clinical consequences, including progressive insulin deficiency, marked glycemic variability, recurrent hypoglycemia, and increased risk of long-term complications. While these consequences can affect all individuals with diabetes, they are particularly consequential in those engaged in physically demanding or safety-sensitive occupations, as in the present case, where glycemic instability may translate into substantial occupational hazards and impaired ability to work safely.

For practitioners, certain clinical features should raise suspicion for LADA, even in older adults. These include lean or normal body habitus, high insulin sensitivity, early or escalating insulin requirements, poor response to oral hypoglycemic agents, unexplained glycemic instability, and coexisting autoimmune disease. In such settings, targeted testing is recommended and includes measurement of islet autoantibodies, glutamic acid decarboxylase (GAD65) antibodies, ICA, insulinoma-associated antigen-2 (IA-2) antibodies, zinc transporter 8 (ZnT8) antibodies, and insulin autoantibodies (IAA), along with assessment of endogenous insulin secretion using C-peptide.

Delayed diagnosis may be particularly consequential in individuals with physically demanding or safety-sensitive occupations, where unpredictable hypoglycemia can pose significant personal and professional risks. This case highlights delayed recognition of LADA in a highly physically active older adult with long-standing diabetes and important occupational implications. Early identification of autoimmune diabetes allows timely initiation of appropriate insulin therapy and access to diabetes technologies such as continuous glucose monitoring (CGM) and automated insulin delivery (AID) systems, which can reduce hypoglycemia and improve glycemic stability.

## Case presentation

A man in his early 70s was referred for endocrine evaluation three years ago because of persistent glycemic instability despite long-standing insulin therapy. He had been diagnosed with presumed T2DM approximately 30 years earlier. His occupation required sustained high-intensity physical activity, necessitating the maintenance of a high level of physical fitness and a low body weight. He reported frequent and unpredictable episodes of both hyperglycemia and hypoglycemia, which caused significant occupational stress and interfered with his ability to work safely.

**Figure 1:**
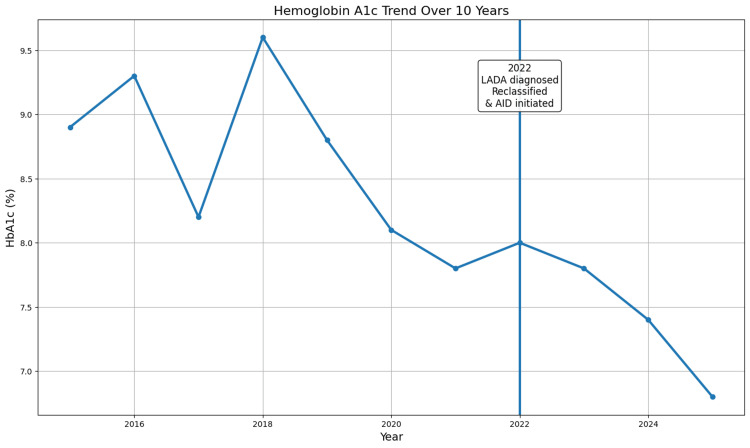
Hemoglobin A1c (HbA1c) trend over 10 years The vertical line at 2022 denotes the time of diagnostic reclassification to latent autoimmune diabetes in adults (LADA) and initiation of automated insulin delivery (AID) therapy. Longitudinal HbA1c values demonstrate prolonged glycemic variability and suboptimal control prior to reclassification, followed by sustained improvement after initiation of advanced insulin therapy. The reference range for HbA1c is 3.8%–5.6%.

In the decade preceding referral, hemoglobin A1c (HbA1c) values remained persistently elevated and variable, ranging from 7.8% to 9.6% (reference range: 3.8%-5.6%), with marked day-to-day glucose fluctuations and failure to achieve stable glycemic control (Figure 1). At presentation, his body mass index (BMI) was 20 kg/m² (reference range: 18.5-24.9 kg/m²). He was using a basal insulin regimen in combination with as-needed short-acting insulin and metformin, but continued to experience significant glucose excursions, including symptomatic hypoglycemia and postprandial hyperglycemia, features considered atypical for insulin-resistant T2DM. He had also previously been treated with empagliflozin, which was discontinued following a diagnosis of bladder cancer prior to presentation to the endocrinology clinic.

Given several atypical features for insulin-resistant T2DM, including a lean body habitus, high insulin sensitivity, long-standing glycemic instability despite insulin therapy, recurrent hypoglycemia, and significant occupational demands, evaluation for autoimmune diabetes was pursued in accordance with consensus recommendations for atypical adult-onset diabetes. Laboratory testing demonstrated markedly elevated GAD65 antibodies (204 IU/mL; reference range <5 IU/mL) and a low fasting C-peptide level (0.54 ng/mL; reference range: 1.10-5.50 ng/mL), confirming LADA with advanced beta-cell failure. Additional evaluation revealed elevated anti-thyroid peroxidase antibodies and hypothyroidism, consistent with coexisting autoimmune thyroid disease, and appropriate treatment was initiated. The overall diagnostic course and therapeutic reclassification are summarized in Table 1.

**Table 1:** Timeline of the cinical course, dagnostic evaluation, and management Reference ranges: HbA1c 3.8–5.6%; body mass index 18.5–24.9 kg/m²; plasma glucose 70–180 mg/dL; hypoglycemia <70 mg/dL; GAD65 antibody <5 IU/mL; C-peptide 1.10–5.50 ng/mL. CGM target mean glucose <154 mg/dL. HbA1c: hemoglobin A1c; T2DM: type 2 diabetes mellitus; GAD65: glutamic acid decarboxylase; LADA: latent autoimmune diabetes in adults; CGM: continuous glucose monitoring

Timeframe	Clinical event	Key finding	Management/outcome
~30 years prior	Initial diagnosis of diabetes mellitus	Presumed type 2 diabetes mellitus	Treated initially with oral hypoglycemic agents
~10 years prior to endocrinology referral	Progressive glycemic deterioration	HbA1c 7.8%–9.6%; marked glycemic variability; lean phenotype (BMI ~20 kg/m²)	Basal insulin initiated; metformin and empagliflozin (later discontinued)
Endocrinology referral (three years prior)	Evaluation for uncontrolled diabetes	Persistent glycemic instability; phenotype atypical for insulin-resistant T2DM	Autoimmune diabetes workup initiated
Diagnostic evaluation	Islet autoantibody testing	GAD65 antibody 204 IU/mL	Diagnosis revised to LADA
	Beta-cell function assessment	C-peptide 0.54 ng/mL	Confirmed insulin-deficient phenotype
Concurrent evaluation	Autoimmune screening	Elevated anti-thyroid peroxidase antibodies	Coexisting autoimmune thyroid disease identified; treatment commenced
Post-diagnosis	Therapeutic reclassification	Insulin-deficient diabetes phenotype	Oral agents discontinued; managed as type 1 diabetes mellitus
Initiation of advanced therapy	Technology-enabled management	Occupational hypoglycemia risk	Tubeless automated insulin delivery with CGM
Longitudinal follow-up	Optimization of insulin therapy	Reduced glycemic variability	Progressive insulin parameter adjustment
Most recent follow-up (Nov 2025)	Stable glycemic control	HbA1c 6.8%; mean glucose 153 mg/dL; time-in-range 74%	Automated mode >90%; no severe hypoglycemia

The diagnosis was revised to LADA, and management was transitioned to a T1DM approach, with discontinuation of oral hypoglycemic agents. The patient received structured education on carbohydrate-based insulin dosing and hypoglycemia prevention, with particular emphasis on insulin adjustment during periods of physical exertion. Given persistent glycemic variability and the occupational risk posed by hypoglycemia, AID therapy was discussed, and the patient elected to initiate a tubeless insulin pump system integrated with continuous glucose monitoring.

Following initiation of AID therapy, basal rates, carbohydrate ratios, and correction factors were progressively individualized. Over longitudinal follow-up, glycemic variability decreased substantially, with marked improvement in overall glycemic control and HbA1c values consistently below 7% (reference range: 3.8%-5.6%). At the most recent follow-up, HbA1c was 6.8%. CGM demonstrated a mean glucose of 153 mg/dL (corresponding to an estimated HbA1c <7%), with 74% time-in-range (70-180 mg/dL) and less than 2% time below range (<70 mg/dL). Automated mode usage exceeded 90%, no severe hypoglycemia was reported, and the patient was able to continue his profession, including periodic out-of-state travel. Comparative glycemic outcomes before and after initiation of AID therapy are summarized in Table 2.

**Table 2:** Glycemic outcomes before and after automated insulin delivery (AID) HbA1c: hemoglobin A1c; CGM: continuous glucose monitoring

Parameter	Before AID	After AID
HbA1c (%); Reference range 3.8%–5.6%	7.8–9.6 (historical)	6.6–6.8
Average glucose (mg/dL) CGM-derived target corresponding to estimated HbA1c <7% (goal <154 mg/dL)	184	153
Glucose variability (%) No single laboratory reference range; assessed using CGM metrics (coefficient of variation <36% considered acceptable)	33	26.5
Time in range (70–180 mg/dL), %	50	74
Time above range (>180 mg/dL), %	35	24
Time very high (>250 mg/dL), %	15	1
Time below range (<70 mg/dL), %	0	<2
Hypoglycemia	Reported	Rare
Glycemic pattern	Marked hyperglycemia with limited time-in-range	Stable glycemia with improved time-in-range
Occupational impact	Glycemic instability interfered with work safety	Able to continue professional work and travel
Quality of life	High burden related to glucose fluctuations	Improved confidence and reduced hypoglycemia-related anxiety

## Discussion

This case illustrates a clinically important scenario in which long-standing T2DM is ultimately reclassified as LADA following recognition of atypical clinical features. LADA should be suspected in adults labeled as having T2DM who exhibit a lean body habitus, marked glycemic variability, early or difficult-to-stabilize insulin requirements, or coexisting autoimmune disease [1,2]. Epidemiologic studies suggest that 5% to 10% of adults initially diagnosed with T2DM may have underlying autoimmune diabetes, underscoring the magnitude of potential misclassification and its clinical implications [2-4].

LADA is frequently underrecognized because early hyperglycemia may respond transiently to noninsulin therapies and because disease onset typically occurs later in life. Fourlanos et al. proposed a clinical screening approach incorporating features such as low BMI and autoimmune history to identify adults who warrant antibody testing [2]. In the present case, decades of presumed T2DM management delayed recognition of autoimmune diabetes, contributing to prolonged glycemic instability, recurrent hypoglycemia, and significant treatment-related frustration. Similar diagnostic delays and clinical trajectories have been reported in prior observational studies and case series, in which reclassification to LADA led to improved glycemic outcomes once insulin-deficiency-focused therapy was initiated [5,6].

High titers of GAD65 antibodies are strongly associated with autoimmune diabetes and predict progression toward insulin dependence [7]. Low C-peptide levels further confirm advanced beta-cell failure and assist in guiding treatment intensity [7]. In this patient, the combination of markedly elevated GAD65 antibodies and suppressed C-peptide supported an insulin-deficient phenotype more consistent with T1DM physiology than T2DM, necessitating a fundamental shift in management strategy.

A particularly important and underemphasized aspect of this case is the impact of delayed diagnosis on quality of life and occupational functioning. The patient’s occupation required sustained physical exertion, variable schedules, frequent travel, and performance of safety-sensitive tasks, all of which increased the risk and consequences of hypoglycemia. Recurrent hypoglycemia generated persistent anxiety, impaired confidence in glycemic self-management, and heightened concern regarding occupational safety. The need to maintain a low body weight further limited the ability to mitigate hypoglycemia through compensatory caloric intake, thereby amplifying glucose variability and psychosocial stress. Collectively, these factors threatened occupational continuity and resulted in a substantial deterioration in quality of life.

Once the diagnosis of LADA was established, management appropriately shifted from nonspecific “type 2” strategies to insulin-deficiency-focused therapy. AID systems have been shown to improve time-in-range and reduce hyperglycemia while maintaining low hypoglycemia exposure in adults with insulin-deficient diabetes [5,6,8]. In this patient, transition to AID resulted not only in objective glycemic improvement but also in restoration of occupational confidence, reduction in hypoglycemia-related anxiety, and improved ability to travel and maintain professional responsibilities, outcomes increasingly recognized as critical endpoints in diabetes care.

**Figure 2:**
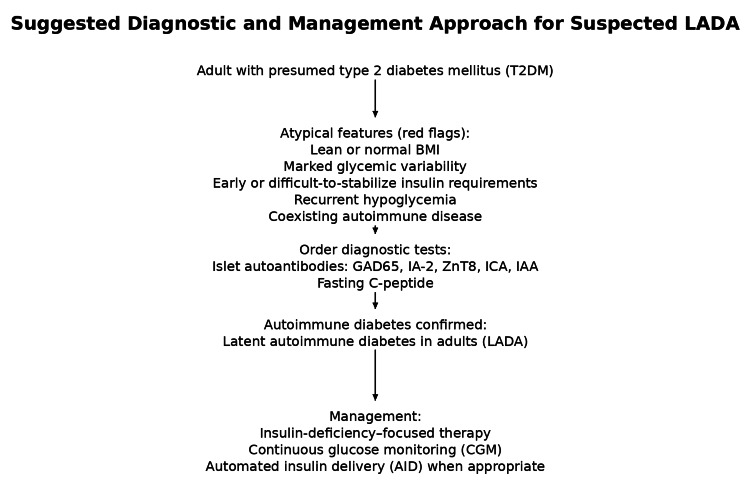
Suggested diagnostic and management approach for suspected latent autoimmune diabetes in adults (LADA) Adults with presumed type 2 diabetes mellitus (T2DM) who exhibit atypical clinical features should undergo islet autoantibody testing (glutamic acid decarboxylase (GAD65), insulinoma-associated antigen-2 (IA-2), zinc transporter 8 (ZnT8), islet cell antibodies (ICA), and insulin autoantibodies (IAA)) and fasting C-peptide measurement. Confirmation of autoimmune diabetes supports reclassification as LADA and transition to insulin-deficiency–focused therapy, including continuous glucose monitoring and automated insulin delivery when appropriate.

LADA is also associated with autoimmune clustering, particularly autoimmune thyroid disease, as demonstrated in this case. Long-term observational data suggest that individuals with LADA experience a time-varying risk of microvascular complications compared with those with T2DM, reinforcing the importance of early recognition, accurate classification, and durable glycemic control [9]. To aid clinicians in recognizing similar presentations, a simplified diagnostic and management approach highlighting key clinical red flags, recommended testing, and treatment considerations is summarized in Figure 2.

This report has limitations inherent to a single-case design, and its findings may not be generalizable to all individuals with adult-onset diabetes. However, the clinical features, diagnostic challenges, and therapeutic response observed here are consistent with patterns reported in the literature and reinforce the importance of maintaining diagnostic vigilance for LADA. This case highlights that timely diagnosis can yield benefits extending beyond glycemic metrics to include functional independence, occupational safety, and overall quality of life.

## Conclusions

LADA should be considered in patients diagnosed with T2DM who exhibit lean body habitus, marked glycemic variability, early insulin dependence, or coexisting autoimmune disease. Epidemiologic evidence indicates that a substantial proportion of adults initially labeled as having T2DM may have underlying autoimmune diabetes. Timely testing with ICAs and C-peptide can accurately reclassify diabetes type and facilitate appropriate, safer therapy. In physically demanding or safety-sensitive occupations, early transition to structured insulin dosing and AID may reduce hypoglycemia risk, improve glycemic stability, and mitigate occupational and psychosocial burden. Importantly, timely diagnosis and technology-enabled management can translate into meaningful improvements in quality of life, including reduced hypoglycemia-related anxiety, greater confidence in daily activities and travel, preservation of functional independence, and the ability to sustain professional roles without compromise.
